# Chaperone co‐inducer BGP‐15 mitigates early contractile dysfunction of the soleus muscle in a rat ICU model

**DOI:** 10.1111/apha.13425

**Published:** 2019-12-18

**Authors:** Nicola Cacciani, Heba Salah, Meishan Li, Hazem Akkad, Anders Backeus, Yvette Hedstrom, Bhanu P. Jena, Jonas Bergquist, Lars Larsson

**Affiliations:** ^1^ Department of Physiology and Pharmacology Karolinska Institutet Stockholm Sweden; ^2^ Department of Physiology Wayne State University School of Medicine Detroit MI USA; ^3^ Analytical Chemistry Department of Chemistry–Biomedical Centre Uppsala University Uppsala Sweden; ^4^ Department of Clinical Neuroscience Clinical Neurophysiology Karolinska Institutet Stockholm Sweden; ^5^ Department of Biobehavioral Health The Pennsylvania State University University Park PA USA; ^6^ Present address: Department of basic medical sciences Faculty of medicine and health sciences An‐Najah National University Nablus Palestine

**Keywords:** immobilization, mechanical ventilation, myopathy, myosin

## Abstract

**Aim:**

Critical illness myopathy (CIM) represents a common consequence of modern intensive care, negatively impacting patient health and significantly increasing health care costs; however, there is no treatment available apart from symptomatic and supportive interventions. The chaperone co‐inducer BGP‐15 has previously been shown to have a positive effect on the diaphragm in rats exposed to the intensive care unit (ICU) condition. In this study, we aim to explore the effects of BGP‐15 on a limb muscle (soleus muscle) in response to the ICU condition.

**Methods:**

Sprague‐Dawley rats were subjected to the ICU condition for 5, 8 and 10 days and compared with untreated sham‐operated controls.

**Results:**

BGP‐15 significantly improved soleus muscle fibre force after 5 days exposure to the ICU condition. This improvement was associated with the protection of myosin from post‐translational myosin modifications, improved mitochondrial structure/biogenesis and reduced the expression of MuRF1 and Fbxo31 E3 ligases. At longer durations (8 and 10 days), BGP‐15 had no protective effect when the hallmark of CIM had become manifest, that is, preferential loss of myosin. Unrelated to the effects on skeletal muscle, BGP‐15 had a strong positive effect on survival compared with untreated animals.

**Conclusions:**

BGP‐15 treatment improved soleus muscle fibre and motor protein function after 5 days exposure to the ICU condition, but not at longer durations (8 and 10 days) when the preferential loss of myosin was manifest. Thus, long‐term CIM interventions targeting limb muscle fibre/myosin force generation capacity need to consider both the post‐translational modifications and the loss of myosin.

## 1 INTRODUCTION

During the past 65 years, intensive care and intensive care units (ICUs) have undergone a significant development related to improvements in medical technology, progress in therapeutics and improved understanding of pathophysiology and pathogenesis. In addition, evidence‐based medicine has resulted in significant changes in the treatment of critically ill ICU patients, moving towards fewer and less invasive interventions and more humane care.^1^ Today, critical care is one of the fastest growing hospital disciplines. Because of considerable improvements in critical care in parallel to the growing need, ICUs have been predicted to occupy one‐third of hospital beds by 2020.^1^ However, life‐saving ICU interventions are also associated with complications with significant negative consequences for morbidity/mortality and health care costs. Approximately 30% of ICU patients who are mechanically ventilated and immobilized for long durations develop a general muscle paralysis of all limb and trunk muscles because of a specific myopathy characterized by an early impact on membrane excitability, excitation‐contraction coupling and followed by a preferential loss of the motor protein myosin, typically referred to as critical illness myopathy (CIM).^2^, ^3^, ^4^ Furthermore, long‐term mechanical ventilation has negative effects on the major inspiratory muscle, the diaphragm, which results in the acquired ventilator‐induced diaphragm dysfunction (VIDD).

Intensive care is one of the most expensive disciplines in modern health care and the impact of CIM and VIDD on patient suffering is enormous. CIM is observed in ~30% of ICU patients who are mechanically ventilated for 5 days and longer, and is associated with threefold higher ICU costs, excluding post‐ICU expenses.^5^ Mechanical ventilation is a life‐saving treatment but weaning from mechanical ventilation is a time‐consuming and expensive process, comprising ~40% of the time spent on the ventilator. In 20%‐30% of these patients, additional problems in weaning are observed resulting in prolonged intensive care with an increased risk of secondary pulmonary complications and mortality because of VIDD. The cost for these patients is staggering and estimated to $64 billion in the US annually, accounting for ~30% of overall ICU costs.^6^ This emphasizes the strong need for research on the mechanisms underlying CIM and VIDD, improved methods for diagnosis/monitoring and the development of efficient intervention strategies targeting the underlying mechanisms.

Mechanistic studies in ICU populations are, however, extremely challenging because of multiple confounding factors impacting on the results such as heterogeneity in age, gender, pharmacological interventions, underlying primary disease and clinical history. There is accordingly a strong need for experimental models including both long‐term mechanical ventilation and immobilization. Unfortunately, commercially available rodent ventilators cannot maintain life support for the long durations required to develop the CIM/VIDD phenotype. In a series of experimental studies using porcine and rat experimental models, not limited by early mortality, we have studied the mechanisms underlying CIM and VIDD at the muscle fibre, motor protein and gene levels.^7^, ^8^, ^9^, ^10^, ^11^, ^12^, ^13^, ^14^, ^15^, ^16^, ^17^, ^18^ A significant loss of muscle mass and force generation capacity (maximum force normalized to fibre size, specific force) was observed in both limb and diaphragm muscles in response to 10 days of mechanical ventilation and immobilization. In both the diaphragm and limb muscles, the loss in specific force was strongly correlated with changes in the motor protein myosin, although the mechanisms were different. In limb muscles, a preferential loss of the motor protein myosin was observed, the hallmark of CIM, while myosin post‐translational modifications hampered myosin function in the diaphragm without a preferential myosin loss.^9^, ^15^, ^19^, ^20^

Heat shock proteins (HSPs) are stress‐induced proteins that are essential for restoring normal cellular function and providing protection from disrupted cell homeostasis. Alterations in HSP protection has been shown to detrimentally impact skeletal muscle structure and function in mechanically ventilated and immobilized animals.^10^, ^12^, ^13^ Furthermore, craniofacial muscles, which are less affected by CIM, show upregulation in HSPs levels, reflecting the protective role of HSPs during ICU conditions. BGP‐15 is a HSP72 co‐inducer that improves insulin sensitivity, increases mitochondrial volume, improves metabolic homeostasis in several rat models of diabetes^21^, ^22^ and improves muscle architecture and contractility in diaphragm muscles in dystrophic mice.^23^ We have previously shown that 10 days BGP‐15 treatment increased the specific force of diaphragm muscle fibres by more than 100%, because of an improved myosin function, compared to untreated muscle fibres in parallel with a partial reversal of post‐translational protein modifications (PTMs).^18^

Given the above outlined differences in muscle specific responses, it cannot be excluded that post‐translational changes in myosin function also contribute to the impaired muscle function in limb muscles prior to the preferential myosin loss. The effects of BGP‐15 treatment on limb (soleus) muscle fibre/myosin function was therefore investigated in animals exposed to the ICU condition (immobilization and mechanical ventilation) for 5, 8 and 10 days and compared with results from the diaphragm after 5 days and previously reported results after 10 days. In both the soleus and the diaphragm, muscle fibre function was impaired after 5 days exposure to the ICU condition and improved in response to BGP‐15 treatment in parallel with protection from myosin PTMs. In the diaphragm, muscle fibre/myosin function was also improved in BGP‐15‐treated animals after 10 days exposure to the ICU condition. In the soleus, on the other hand, BGP‐15 did not reduce the preferential myosin loss after 8 and 10 days or improve soleus muscle fibre function. Thus, interventions targeting the impaired limb muscle fibre function in patients with CIM need to address both the impaired muscle fibre/myosin function and myosin loss.

## 2 RESULTS

### 2.1 Animals and survival

**Figure 1:**
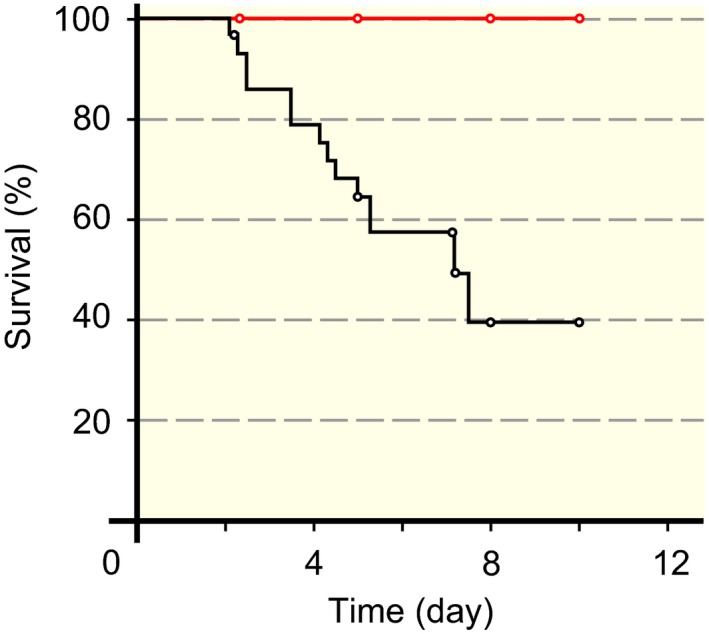
Survival in control and BGP‐15‐treated animals. Kaplan‐Meier survival plot showing 100% survival of the 13 BGP‐15‐treated animals killed at 5, 8 and 10 d. The corresponding survivals in control animals (n = 30) were 67% (5 d), and 39% (8‐10 d)

Adult female Sprague‐Dawley rats were included in this study to investigate the effects of BGP‐15 treatment on soleus and diaphragm muscles during 5, 8 and 10 days of exposure to the ICU condition (immobilization and mechanical ventilation). During the initial 48 hours of the experiments, mortality is primarily related to technical problems related to instrumentation or complications during initial surgery. Survival has therefore been monitored after the initial 48 hours of the experimental period. A dramatic improvement in survival was observed in animals treated with BGP‐15 compared with control animals. In control animals (n = 30), 67% of the animals survived 5 days and 39% survived up to 10 days exposure to the ICU condition. The most common cause underlying mortality was a mucous plug not removed quickly enough during the 24 h/d monitoring. In animals treated with BGP‐15 (n = 13), 100% survival was observed after 5, 8, and 10 days exposure to the ICU condition. In controls, we have also included rats exposed to the ICU condition at other durations than 5, 8 and 10 days, that is, results included from previous studies.^9^, ^24^ The logrank statistic showed a significant (*P* < .01) survival benefit with BGP‐15 (Figure 1).

### 2.2 The effects of BGP‐15 treatment on body weight, muscle size and myosin expression

**Figure 2:**
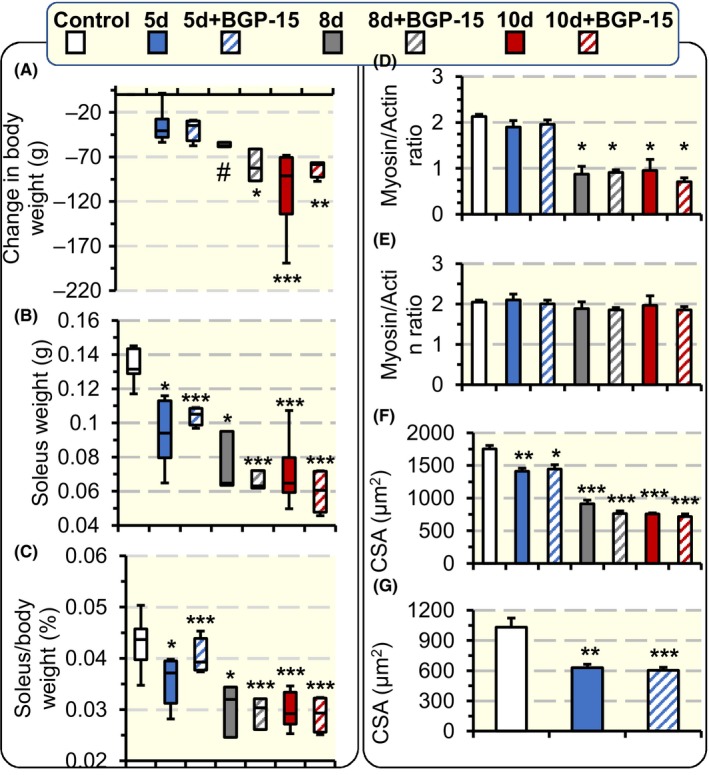
Rats body weight, soleus muscle weight, Myosin: actin ratio, and single muscle fibre CSA. The change in rats body weight (A), soleus muscle weight (B) and the per cent of soleus to body weight change (C) in controls and rats exposed to the ICU condition for 5, 8 and 10 d and rats exposed to BGP‐15 treatment and the ICU condition for 5, 8 and 10 d. Myosin: actin ratios in the soleus (control n = 5, 5d n = 3, 5d + BGP‐15 n = 4, 8d n = 3, 8d + BGP‐15 n = 3, 10d n = 5, and 10d + BGP‐15 n = 3) (D), and in the diaphragm (control n = 4, 5d n = 5, 5d + BGP‐15 n = 2, 8d n = 2, 8d + BGP‐15 n = 2, 10d n = 4, and 10d + BGP‐15 n = 2) (E) determined by 12% SDS‐PAGE, with the dots on the figure represent the individual ratio from each rat. Single muscle fibre CSA in the soleus (F) and in the diaphragm (G) measured at fixed sarcomeric length. In all the figure panels, the p values represent statistical significance when compared with control except when stated otherwise in the figure. Values are represented as means + SEM. *P < .05; **P < .01; ***P < .001

Independent of BGP‐15 treatment, body weights decreased 13%, 22% and 31% during exposure to the ICU condition for 5, 8 and 10 days, respectively, albeit statistically significant only in the 10 days group (*P* < .001; Figure 2A). Soleus muscle weight was reduced (*P* < .05‐.001) 27%, 47% and 51% after 5, 8 and 10 days exposure to the ICU condition respectively (Figure 2B). The soleus to body weight ratio was reduced after 5 days of exposure to the ICU condition and remained reduced at 10 days exposure to the ICU condition (*P* < .001; Figure 2C) indicating a more severe soleus than body weight loss. In accordance with our previous observations in the soleus and diaphragm,^19^, ^25^ the preferential myosin loss, the hallmark of CIM in ICU patients, appeared first at durations longer than 5 days exposure to the ICU intervention (*P* < .05; Figure 2D), while the myosin: actin ratio was not affected in the diaphragm in response to different durations of the ICU condition (Figure 2E). In addition, BGP‐15 treatment had no significant effect on the myosin: actin ratio when compared to untreated groups in either soleus or diaphragm (Figure 2D,E). The cross‐sectional areas (CSAs) of the myofibres isolated from the soleus and diaphragm were reduced in response to the ICU condition; in the soleus CSAs decreased (*P* < .001) 19%, 52% and 58% after 5, 8 and 10 days of exposure to the ICU condition, and in the diaphragm CSAs decreased (*P* < .001) 40% after 5 days of exposure to the ICU condition regardless of BGP‐15 treatment (*P* < .001; Figure 2F,G).

### 2.3 The effect of BGP‐15 on regulation of muscle contraction

**Figure 3:**
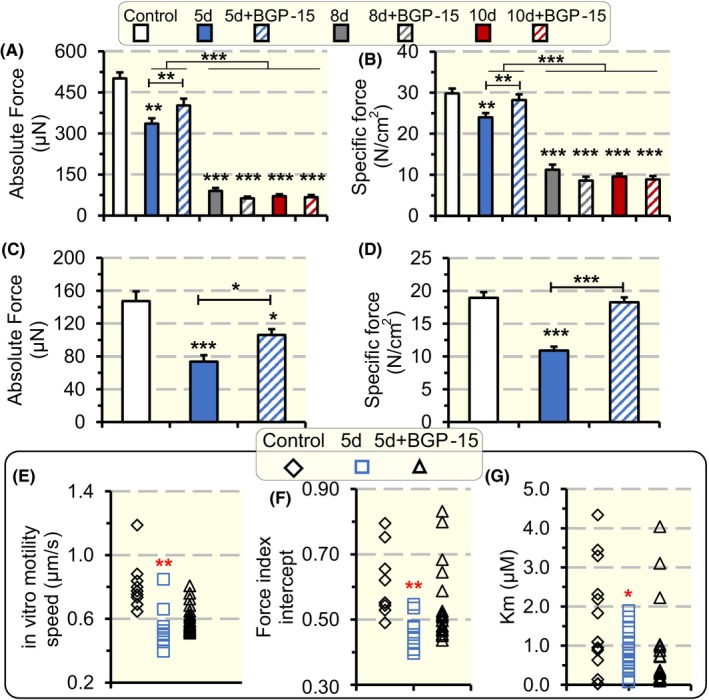
Single muscle fibres absolute force, specific force and myosin in vitro motility speed and force. Single muscle fibre absolute force measured at fixed sarcomeric length (A), and specific force (B) in soleus fibres in response to exposure to the ICU condition and BGP‐15 treatment for 5, 8 and 10 d. Single muscle fibre absolute force measured at fixed sarcomeric length (C), and specific force (D) in diaphragm fibres in response to exposure to the ICU condition and BGP‐15 treatment for 5 d. In vitro motility speed (E), force index (F) and efficiency (G) of myosin extracted from soleus single muscle fibre segments expressing the type I MyHC in rats exposed to the ICU condition for 5 d with and without BGP‐15 treatment. Values are represented as means + SEM. *P < .05; **P < .01; ***P < .001

In the soleus, a total of 304 soleus fibres met the acceptance criteria and were included in the study after 5, 8 and 10 days exposure to the ICU condition. Five days exposure to the ICU condition reduced single muscle fibre maximum absolute force and maximum force normalized to muscle fibre CSA (specific force) compared with control fibres (*P* < .01; Figure 3A,B), but not in animals treated with BGP‐15. Exposure to the ICU condition for 8 and 10 days, on the other hand, reduced muscle fibre maximum absolute force and specific force regardless of BGP‐15 treatment (*P* < .001; Figure 3A,B).

In the diaphragm, a total of 164 single fibres met the criteria for acceptance after 5 days exposure to the ICU condition. Both absolute force and specific force were reduced after 5 days of exposure to the ICU condition (*P* < .001; Figure 3C,D) while BGP‐15 treatment for 5 days increased absolute and specific force of the diaphragm muscle fibres (*P* < .05‐.001; Figure 3C,D) in accordance with previous observations after 10 days exposure to the ICU condition.^18^

To detect if the improvement in specific force in the soleus during 5 days exposure to the ICU condition and BGP‐15 treatment was related to changes in myosin function, myosin was extracted from soleus muscle fibres and the catalytic properties (motility speed), and force generation capacity of myosin was investigated using a single muscle fibre in vitro motility assay.^26^, ^27^ In addition, the affinity (Km) of the myosin ATPase to its substrate ATP was measured from myosin extracted from single muscle fibre segments. Exposure to the ICU condition for 5 days reduced motility speed (*P* < .01‐.001) in both untreated and BGP‐15 treated animals (Figure 3E). Myosin force generation capacity, on the other hand, decreased (*P* < .01) in the untreated animals, but not in the BGP‐15 treated rats, after 5 days exposure to the ICU condition while the affinity of myosin ATPase for ATP increased as indicated by decreased Km (*P* = .05;Figure 3F,G). All soleus single muscle fibre in vitro motility assays were performed in muscle fibres expressing the dominant myosin heavy chain (MyHC) isoform in the rat soleus, that is, the β/slow (type I) MyHC isoform.

We have previously shown that 10 days exposure to the ICU condition has a severe negative effect on diaphragm muscle fibre specific force, but treatment with BGP‐15 increased the diaphragm muscle fibre specific force ~100% compared to rats exposed to the ICU condition without BGP‐15 treatment, because of reduced myosin PTMs and improved myosin force generation capacity.^18^ In the soleus, a similar effect was observed after 5 days exposure to the ICU condition, but not at longer durations when a preferential myosin loss was observed. In an attempt to improve our understanding of the mechanisms underlying the effects of the ICU condition and the BGP‐15 intervention, mass spectrometry was used to measure myosin PTMs.

### 2.4 Mass spectrometry driven proteomics

The sequence coverage of the target protein, the β/slow type I myosin isoform (*Myh‐7)* did not differ between control (59% ± 1%), 5‐day ventilated (62% ± 2%) and 5‐day ventilated with BGP‐15 treatment (61% ± 1%). A large number of PTMs were observed in the type I myosin isoform in controls, 5 days mechanically ventilated and 5‐days mechanically ventilated together with BGP‐15 treatment. We have focused on modifications induced by 5 days of mechanical ventilation with or without the BGP‐15 intervention and the criteria for selecting these PTMs were: (a) the presence in ≥75% of all mechanically ventilated animals; (b) the PTM should be either absent or present in <25% of the control group animals; (c) modifications should be verified by MS/MS data; and (d) oxidized methionines were excluded since they may represent artefacts from sample preparation. Several modifications were identified but could not be fully localized to a specific amino acid location in the targeted sequence region. Those modifications were not included in the final results.

**Figure 4:**
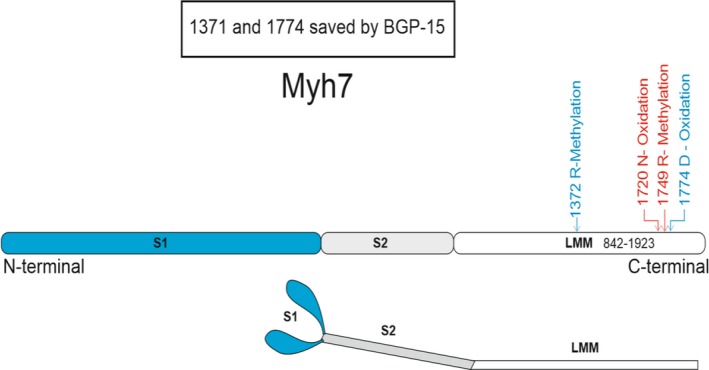
β/slow (type I) MyHC post‐translational modifications. The BGP‐15 treatment was found to rescue the myosin motor protein from two PTMs (labelled in blue), that is, a methylation in the 1372 position (arginine) and oxidation in the 1774 position (aspartic acid). At the 1720 position (asparagine) we found an oxidation and in the 1749 position (arginine) and a methylation independent on BGP‐15 in response to 5 d mechanical ventilation (labelled in red)

The BGP‐15 treatment was found to protect the myosin motor protein from two PTMs, both located in the rod region, that is, a methylation in the 1372 position (arginine) and oxidation in the 1774 position (aspartic acid). Methylation of arginine will substantially increase the hydrophobicity over the whole molecule and decrease the charge while oxidation of aspartic acid will neither change charge nor hydrophobicity. Furthermore, at the 1720 position (asparagine) we found an oxidation and in the 1749 position (arginine) a methylation independent on BGP‐15 in response to 5 days mechanical ventilation (Table 1, Figure 4).

**Table 1:** Post‐translational modifications of myosin in response to long‐term mechanical and immobilization with and without BGP‐15 treatment

Group comparison	Modification, position	Found in	Confirmation MS/MS^a^,^b^
Group ‘5 days, no drug' vs control group (in all 5 days, no drug but also in 1‐2 of the controls)	Acetylation, 83 K	2 out of 6 control samples	NO
	Methylation, 1372 R	1 out of 6 control samples	YES
	Methylation, 1749 R	2 out of 6 control samples	YES
	Oxidation, 826 N	2 out of 6 control samples	NO
	Oxidation, 1720 N	2 out of 6 control samples	YES
Group ‘5 days, no drug' vs group ‘5 days + BGP‐15' (in all 5 days, no drug but also in 1 of 5 days, BGP‐15 treated)	Methylation, 1372 R	1 out of 4 samples of group ‘5 days + BGP‐15'	YES
	Oxidation, 826 N	1 out of 4 samples of group ‘5 days + BGP‐15'	NO
	Oxidation, 1771 K	1 out of 4 samples of group ‘5 days + BGP‐15'	NO
	Oxidation, 1774 D	1 out of 4 samples of group ‘5 days + BGP‐15'	YES
Group ‘5 days + BGP‐15' vs control group (in all 5 days + BGP‐15 but also in 1‐2 of controls except when unique)	Acetylation, 72 K	1 out of 6 control samples	NO
	Methylation, 1749 R	2 out of 6 control samples	YES
	Oxidation, 490 N	Unique	YES
	Oxidation, 1234 N	1 out of 6 control samples	NO
Group ‘5 days + BGP‐15' vs group ‘5 days, no drug'	Acetylation, 680 S	Unique	NO

a Oxidation, 1774 D, Oxidation, 490 N were confirmed using MaxQuant.

b Oxidation, 1720 N was confirmed using both MaxQuant and PD.

### 2.5 The effect of BGP‐15 on transcriptional regulation of protein synthesis and degradation

**Figure 5:**
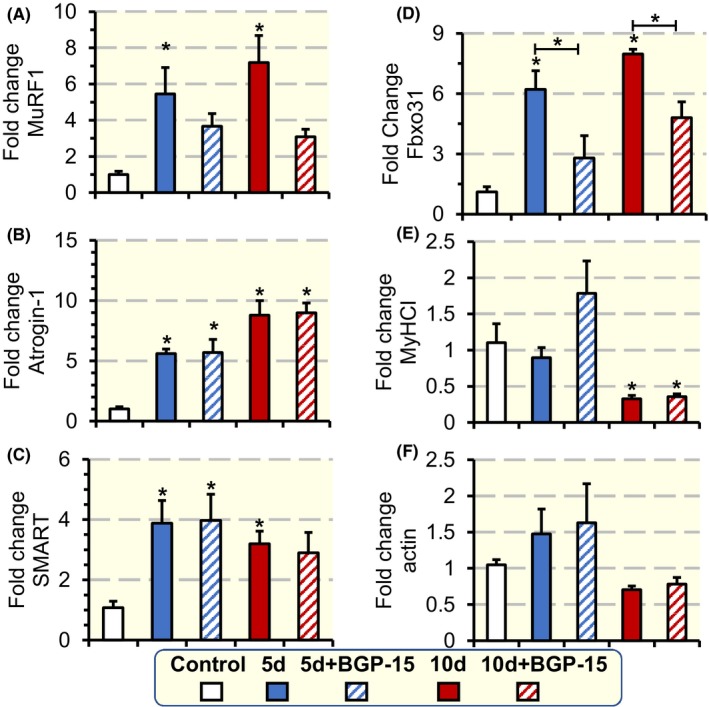
mRNA expression of E3 ligases, MyHCI isoform and actin in the soleus. mRNA expression of the E3 ligases MuRF1 (A), Atrogin‐1(B), SMART (C) and Fbxo31 (D), and MyHCI (E), and actin (F) in the soleus of rats exposed to the ICU condition and BGP‐15 treatment for 5 and 10 d. For each experimental group the values were normalized to the controls. Values are presented as means + SEM. Significant differences compared with controls are denoted as *P < .05

In the ubiquitin‐proteasome system, ubiquitin targets proteins to be identified for degradation,^28^ while ubiquitin ligase enzymes (E3) bind proteins and assist in their ubiquitination, which unfold the proteins and feed them into the proteasome in an ATP‐dependent manner.^29^ In the soleus, exposure to the ICU condition for 5 and 10 days increased the gene expression of the E3 ligases MuRF1 (*P* < .05; Figure 5A), Atrogin‐1 (*P* < .05; Figure 5B), SMART (*P* < .05; Figure 5C) and Fbxo31 (*P* < .05; Figure 5D). In the BGP‐15‐treated animals, on the other hand, there was no upregulation of MuRF1 and Fbxo31 expression after 5 and 10 days of exposure to the ICU condition (Figure 5A,D). However, BGP‐15 did not influence the upregulation of Atrogin‐1 and SMART after 5 and 10 days of exposure to the ICU condition (Figure 5B,C). In addition, the gene expression of the dominant myosin isoform in the soleus, that is, myosin heavy chain I (MyHCI; Figure 5E), was not affected after 5 days but was significantly reduced (*P* < .05) after 10 days of exposure to the ICU condition regardless of BGP‐15 treatment. The gene expression of actin was not significantly affected by the ICU condition or BGP‐15 treatment (Figure 5F).

**Figure 6:**
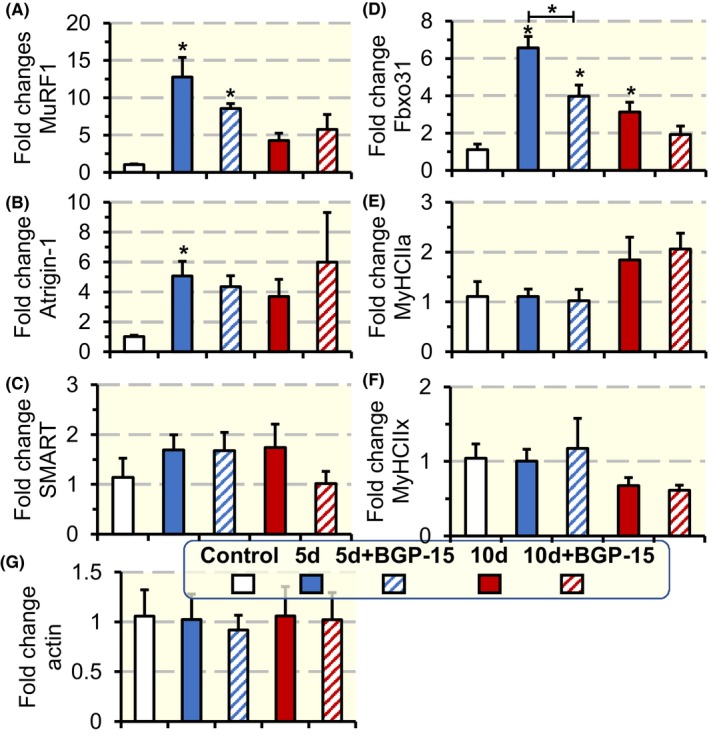
mRNA expression of E3 ligases, MyHCs isoforms and actin in the diaphragm. mRNA expression of the E3 ligases MuRF1 (A), Atrogin‐1 (B), SMART (C) and Fbxo31 (D); myosin heavy chain isoforms MyHCIIa (E), MyHCIIx (F); and actin (G) in the diaphragm muscles of rats exposed to the ICU condition and BGP‐15 treatment for 5 and 10 d. The values for the different experimental groups were normalized to the control group. Values are presented as means + SEM. Significant differences compared with controls are denoted as *P < .05

In the diaphragm, an increase in the gene expression of the E3 ligases MuRF1 (*P* < .05; Figure 6A) and Atrogin‐1 (*P* < .05; Figure 6B) was observed after 5 days exposure to the ICU condition. The SMART gene expression was not affected (Figure 6C), while Fbxo31 gene expression was increased after both 5 and 10 days exposure to the ICU condition (*P* < .05; Figure 6D). BGP‐15 administration did not affect the expression of these E3 ligases when compared to untreated rats except for Fbxo31 expression which was reduced after 5 days BGP‐15 treatment when compared with untreated rats (*P* < .05; Figure 6D). Actin and MyHC isoform expressions were not affected by the ICU condition with or without BGP‐15 treatment Figure 6E‐G).

### 2.6 The effect of BGP‐15 on contractile protein gene expression and mitochondrial morphology, dynamics and biogenesis

The gene expression of the dominant myosin isoform in the soleus, that is, β/slow myosin heavy chain isoform (type I), was not affected after 5 days but was significantly reduced (*P* < .05) at the longer durations of exposure to the ICU condition regardless of BGP‐15 treatment. The gene expression of actin was, on the other hand, not significantly affected in the limb muscle by the ICU condition or BGP‐15 treatment. In the diaphragm, actin and MyHC isoform expression were not affected by the ICU condition with or without BGP‐15 treatment.

**Figure 7:**
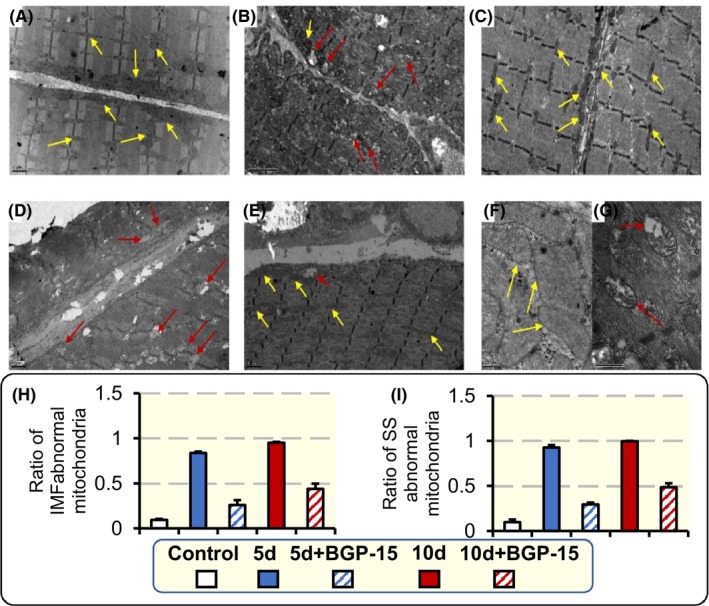
Intermyofibrillar (IMF) and subsarcolemmal (SS) mitochondria structural changes. Electron micrographs captured at 10 000× magnification showing IMF and SS mitochondria structure in the soleus muscles of control animals (A), animal exposed to the ICU condition for 5 d (B), and 10 d (D), and animals treated with BGP‐15 and exposed to the ICU condition for 5 (C) and 10 d (E). Mitochondria structure at 40 000× magnification is shown in control animals (F), and animals exposed to 10 d ICU treatment (G). Ratio of IMF mitochondria abnormal fraction for all animal groups (H). Ratio of SS mitochondria abnormal fraction for all animal groups (I). Examples of normal mitochondria structure, with electron dense matrix and organized cristae, are labelled with yellow arrows, while some abnormal mitochondria, with swollen or vesicular structure, are labelled with red arrows. Values are presented as means + SEM

In the soleus, the structural changes in mitochondria were analysed using transmission electron microscopy in a subsample of the animals. Intermyofibrillar (IMF) mitochondria, 91% showed a normal structure in control animals, that is, well‐defined borders, high electron density matrix and well‐organized cristae (Figure 7A,F). The ICU condition induced dramatic changes in IMF mitochondrial morphology that varied according to the duration of immobility (Figure 7B,D,H). Normal fractions were only observed in 16% and 5% of the IMF mitochondria in the 5 and 10 days groups respectively. In these groups, more vesicular or swollen mitochondria were observed with less organized cristae, and less electron‐dense matrix (Figure 7G). BGP‐15 treatment in 5d + BGP‐15, and 10d + BGP‐15 groups showed an improvement in IMF mitochondria structure, that is, normal fractions were observed in 75% and 56% of mitochondria in the 5 and 10 days groups respectively. Thus, BGP‐15 had a protective effect on mitochondria structure in animals exposed to the ICU condition, although it should be emphasized that these analyses were restricted to a subsample of the studied animals (Figure 7C,E,H).

In subsarcolemmal (SS) mitochondria, 90% showed normal morphology in control animals (Figure 7A,F). Exposure to the ICU condition increased the abnormal ratio of SS mitochondria to 93%, and 98% after 5 and 10 days respectively (Figure 7B,D,I). In rats administered BGP‐15, 29% and 48% abnormal SS mitochondria were observed after 5 and 10 days, respectively, indicating that the drug decreased the negative structural changes observed in response to ICU intervention also in the SS region (Figure 7C,E,I).

**Figure 8:**
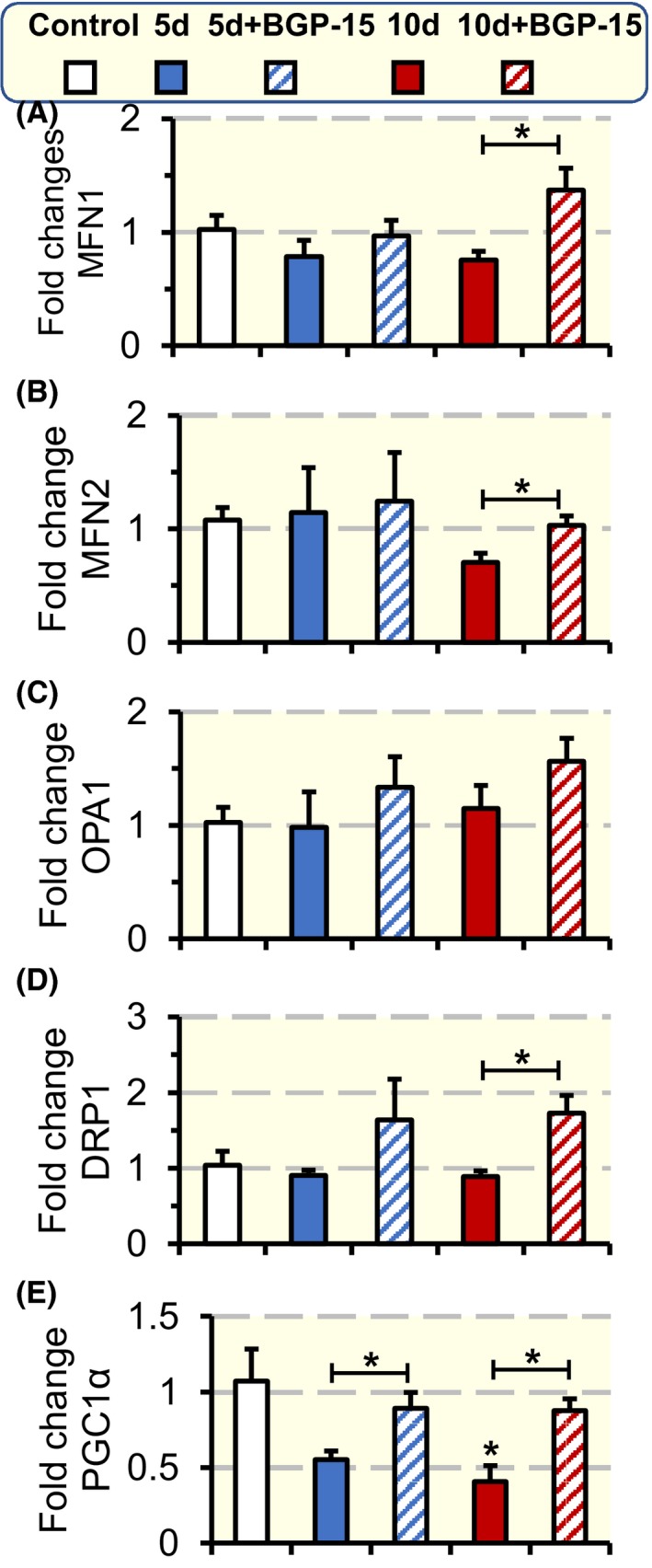
Mitochondrial dynamic and biogenesis. Mitochondrial dynamic gene expression (A‐D); mitochondrial fusion gene expression MFN1 (A), MFN2 (B), and OPA1 (C), and mitochondrial fission gene expression DRP1 (D). Mitochondrial biogenesis evaluated as the gene expression of total PGC‐1α (E) for each experimental group normalized to control group. Values are presented as means + SEM. Significant differences compared with controls are denoted as *P < .05

To investigate the mechanisms underlying the structural changes observed in mitochondria, markers for mitochondrial dynamics and biogenesis were studied in the soleus muscle. The gene expression of mitochondrial fusion (Mitofusin 1 [MFN1], Mitofusin 2 [MFN2] and Optic atrophy 1 [OPA1]) and mitochondrial fission dynamin‐related protein 1 (DRP1) markers was measured. Mitochondrial dynamics markers were not affected by 5 and 10 days exposure to the ICU condition (Figure 8A‐D). However, BGP‐15 administration for 10 days increased MFN1, MFN2 and DRP1 levels (*P* < .05; Figure 8A,B,D respectively) compared with 10 days untreated rats. Mitochondrial biogenesis was evaluated by measuring the levels of the transcriptional coactivator peroxisome proliferator‐activated receptor γ coactivator 1‐α (PGC‐1α). Ten days exposure to the ICU condition reduced PGC‐1α levels (*P* < .05; Figure 8E), indicating an impaired mitochondrial biogenesis. On the other hand, BGP‐15 treatment for 5 and 10 days, significantly elevated PGC‐1α levels (*P* < .05; Figure 8E) compared with untreated animals, indicating an improved mitochondrial biogenesis in response to BGP‐15 treatment.

## 3 DISCUSSION

We have previously shown that the chaperone co‐inducer BGP‐15 positively impacts diaphragm muscle fibre force generation capacity during long‐term (10 days) mechanical ventilation by protecting the molecular motor protein myosin from post‐translational modifications that hinder myosin function.^18^ In the current study, we have extended these analyses to examine the effects of the BGP‐15 intervention on survival and its impact on a distal hindlimb muscle during 5, 8 and 10 days mechanical ventilation. In both the soleus and diaphragm, 5 days mechanical ventilation and immobilization were associated with a decline in muscle fibre force generation capacity (specific force) which was mitigated by BGP‐15 treatment. In the diaphragm, the relative loss in specific force at 5 days was less than in our previous study after 10 days mechanical ventilation,^18^ suggesting an association between the decline in diaphragm function, the duration of the ICU condition and progressive post‐translational modifications of contractile proteins. In contrast to the diaphragm, there is a progressive preferential myosin loss in the soleus in response to mechanical ventilation and immobilization for durations longer than 5 days.^9^, ^15^ BGP‐15 treatment did not affect the preferential myosin loss in the soleus, which becomes the dominant factor underlying the decrease in specific force at longer durations. Thus, efficient interventions targeting impaired contractile protein function associated with CIM need to target both myosin modifications negatively affecting myosin function as well as the myosin loss.

An unexpected, but potentially clinically relevant observation was the significant improvement in survival in BGP‐15‐treated animals compared with untreated controls. BGP‐15 belongs to an emerging class of ‘membrane‐lipid therapy' pharmaceuticals which possess the ability to enhance stress tolerance, but their precise mechanisms are not fully known.^30^ The chaperone co‐inducer BGP‐15 upregulates HSP synthesis via multiple other mechanisms: (a) inhibition of PARP‐1 activity, (b) membrane stabilizing effects by preserving the overall order of the lipid bilayer in cells, activating the membrane‐associated signalling molecule Rac1 which increases HSF‐1 and HSP synthesis,^30^ and (c) activation of the respiratory chain, inhibition of free radical production and protection of mitochondria against toxicity, which in turn increase mitochondrial integrity.^30^

Janus kinase (JAK) signalling has been identified as a key trigger mechanism upstream of signal transducer in the diaphragm in rats exposed to controlled mechanical ventilation (CMV).^31^, ^32^ In a more recent study, the temporal activation of the JAK/STAT3 pathway was investigated in the current experimental model at durations varying from 6 hours to 14 days.^33^ The activation of the JAK/STAT3 pathway was confirmed in the diaphragm as well as in limb and intercostal muscles, but the temporal pattern of activation varied between the different muscles.^34^ Furthermore, interleukin 6 (IL‐6) receptors and IL‐6 signal transducers interacting with JAKs to elicit intracellular signalling were upregulated in response to the ICU condition in the three investigated muscles.^34^ This is of specific interest since the JAK/STAT3 pathway has been reported to be involved in the muscle wasting induced by IL‐6 in cancer cachexia.^35^ In rats exposed to the ICU condition for 7‐8 days (n = 9), unpublished results from our group have shown an improved 90% survival in rats treated with a JAK/STAT inhibitor (ruxolitinib). Furthermore, pilot results have shown an inhibition of visceral adipocyte IL‐6 production in rats treated with BGP‐15 during exposure to the ICU condition. Thus, it may be speculated that the anti‐inflammatory effect of BGP‐15 via the IL‐6 activation of JAK/STAT3 pathway contributes to the improved survival in rats exposed to the ICU condition. This is supported by the improved survival in response to the treatment with a dissociative glucocorticoid (Vamorolone) derived from prednisolone in rats exposed to the ICU condition for 5 days.^36^, ^37^, ^38^ Vamorolone has similar anti‐inflammatory effects as prednisolone, but without the harsh negative side effects of prednisolone on skeletal muscle, such as accelerate protein degradation exacerbating muscle wasting.

We have previously shown that distal hindlimb muscles respond with atrophy, decreased specific force and preferential myosin loss independent of fast, mixed or slow‐twitch type. Although a slight trend of atrophy and myosin loss being more prominent in slow‐ than fast‐twitch muscles and interpreted to be secondary to a faster protein turnover rate in slow‐ than fast‐twitch muscles.^9^ However, the mechanisms underlying the impaired force generation capacity (specific force) at the single muscle fibre level differ between limb and diaphragm muscles, although the molecular motor protein myosin plays a critical role in both limb and respiratory muscles. In limb muscles, the preferential myosin loss is the dominant factor underlying the decreased specific force during long‐term mechanical ventilation. In the diaphragm, X‐ray diffraction, mass spectrometry and immunoblotting analyses have shown that myosin function hampered by post‐translational modifications play an integral role for the impaired force generation capacity of diaphragm muscle fibres and myosin in response to the ICU condition.^18^, ^25^ The 56% increase in diaphragm muscle fibre specific force during 5 days exposure to the ICU condition and BGP‐15 treatment, corresponding to ~100% of control values, is in accordance with our previous observations of a ~100% increased specific force by BGP‐15 treatment during 10 days exposure to the ICU condition, corresponding to 75% of control.^18^ According to immunoblotting, mass spectrometry and single fibre in vitro motility measurements, the improved specific force was primarily because of the protection of myosin from post‐translational modifications and maintained function of the molecular motor protein myosin.^18^ The improved absolute (20%) and specific force (18%) in soleus fibres treated with BGP‐15 prior to the myosin loss after 5 days exposure to the ICU condition was related to a similar mechanism as in the diaphragm, that is, an improved myosin function related to protection of myosin from modifications. After 10 days mechanical ventilation, the diaphragm was protected from 28 different modifications by BGP‐15 treatment with the large majority in the rod region (25 of 28). Fewer mechanical ventilation and immobilization induced myosin modifications were detected in response to BGP‐15 treatment in the soleus, but it is interesting to note that two of the affected modifications in the diaphragm after 10 days mechanical ventilation were observed in the same rod region as in the soleus, that is, nitration of tryptophan in position 1375 and deamidation of glutamine in the 1777 position. Although the sequence coverage of the target myosin is 60% and other significant modifications affecting the regulation of muscle contraction may go undetected, it is of specific interest that the impaired muscle function at the muscle cell and motor protein levels associated with ageing and VIDD was also associated with myosin rod modifications in the soleus after 5 days of exposure to the ICU condition.^18^, ^39^ Furthermore, mutations in the rod region of the type I myosin isoform in the 1729 position are associated with progressive muscle weakness and atrophy in patients as well as in experimental models.^40^, ^41^, ^42^ The molecular structure of the rod region of myosin is largely unknown, but it is becoming increasingly evident that the rod region has functions which go beyond being a simple scaffold to present the head domain to actin and to transmit force.^40^ Thus, the packing of the myosin rod region in the thick filament backbone may impact on the structure of the myosin head and regulation of muscle contraction presumably related to the recruitment of myosin heads during force generation via mechanosensation^43^, ^44^ and unrelated to Ca^2+^ thin filament activation.^40^

BGP‐15 treatment did not have an impact on the progressive myosin loss observed at durations longer than 5 days. Hence, the mechanisms underlying the loss in muscle force at the motor protein level in response to long‐term exposure to the ICU condition appear to be related to an initial post‐translational modification of myosin and followed by a myosin loss and where the former mechanism is alleviated by BGP‐15 treatment. The activation of the ubiquitin‐proteasome protein degradation system has been shown to be closely associated with the muscle wasting and preferential myosin loss associated with CIM.^9^, ^14^ Activation of E3 ligases in response to the ICU condition was confirmed in the current study, but the blunting of the MuRF1 and Fbxo31 upregulation in response to BGP‐15 treatment in animals exposed to the ICU condition did not impact on the muscle wasting, preferential myosin loss or the transcription downregulation of myosin in the soleus muscle. This demonstrates the complexity of transcriptional activation of the ubiquitin‐proteasome degradation pathway and the interaction between different E3 ligases as well as the need for further studies both at the gene and protein levels.

In an attempt to understand the observed effects of ICU intervention and BGP‐15 treatment on the soleus muscle, we evaluated mitochondrial structure, and several dynamics and biogenesis markers. The skeletal muscle tissue has two subpopulations of mitochondria: IMF mitochondria that are positioned among myofibrils and SS mitochondria.^45^ IMF usually accounts for almost 80% of skeletal muscles mitochondria. IMF and SS differ in their protein content and function. For instance, IMF expresses higher levels of OxPhos protein complexes, and have superior mitochondrial coupling, and thus, IMF is highly specialized in calcium handling and energy production for contractile activity. SS mitochondria provide energy for membrane‐related events. In our study, we analysed the effects on these subpopulations separately, but our results showed that there was no difference between these two subpopulations in response to the CIM intervention or BGP‐15 treatment.

In the soleus, the mitochondrial morphology, mitochondrial dynamics (gene expression of MFN1, MFN2 and DRP1) and biogenesis (PGC‐1α levels) were positively affected by BGP‐15 treatment. The increase in PGC‐1α in response to 5 and 10 days of BGP‐15 treatment is of specific interest as PGC‐1α is a transcriptional coactivator that binds a large complement of transcription factors that are directly associated with mitochondrial respiratory function or act directly on the expression of the respiratory chain.^46^ In addition, the increase in PGC‐1α by BGP‐15 is important not only for increasing the mitochondrial mass in the muscle and thus increasing ATP supply to prevent bioenergetic crises, but also for improving mitochondria maintenance by continuously providing newly synthesized mitochondrial components. Furthermore, mitochondrial maintenance is further improved by BGP‐15 treatment through its effects on improving mitochondrial dynamics.^47^ As shown in the soleus muscle, the improved mitochondrial maintenance will reduce ROS production and protect against mitochondrial structural damage.

Other mechanisms involved in the major structural abnormalities observed in this study may relate to mitophagy. In a previous study performed by our group, Bnip3, Pink1 and Parkin were analysed at the transcriptional level to study the effects on the selective removal of damaged mitochondria via autophagy, termed mitophagy, in response to the ICU intervention and passive mechanical loading. The study showed that Parkin, Pink1 and Bnip3 levels were upregulated on the unloaded side after 9‐14 days of the ICU intervention.^14^ Thus, the improved mitochondrial structure/function in the soleus is accordingly anticipated to reduce oxidative stress and protein modifications as confirmed by Raman spectroscopy analyses.

In conclusion, BGP‐15 has a strong effect on survival, a significant and persistent effect on diaphragm muscle fibre function, and a significant albeit temporary positive effect on limb single muscle fibre and motor protein force generation capacity in animals exposed to long‐term mechanical ventilation and immobilization. BGP‐15 treatment protects myosin from post‐translational modifications hampering myosin and muscle fibre function, presumably at least in part by maintaining improved mitochondrial structure and biogenesis and reducing the levels of the E3 ligases MuRF1 and Fbxo31. Thus, BGP‐15 offers a novel intervention strategy aiming at reducing the negative effects of life‐saving interventions in critically ill ICU patients.

## 4 MATERIALS AND METHODS

### 4.1 Animals and ICU model

All experimental groups were deeply sedated with isoflurane, mechanically ventilated and pharmacologically paralysed post‐synaptically with alpha cobrotoxin. Thirty‐six adult female Sprague‐Dawley rats were divided into one sham‐operated control group (n = 10) and the six experimental groups exposed to deep sedation, CMV, neuromuscular blockade (NMB) for 5 days with BGP‐15 treatment (5d + BGP‐15, n = 4) and without BGP‐15 (5d, n = 6), 8 days with BGP‐15 treatment (8d + BGP‐15, n = 3) and without BGP‐15 treatment (8d, n = 3), and 10 days with BGP‐15 (10d + BGP‐15, n = 4) and without BGP‐15 (10d, n = 6). For survival data in control rats, we also included results from other rats exposed to the ICU condition at other durations than 5, 8 and 10 days longer than 2 days (total number of controls, 30) included in previous studies.^9^, ^24^

All experimental animals were maintained in fluid and nutritional balance throughout the duration of the experimental procedures by introducing (a) intra‐arterial solution (0.6 mL/h) containing 21 mL H_2_O, 24 mL 0.5 N lactated Ringer, 0.84 g oxacillin Na, 0.65 mg alpha‐cobrotoxin, 0.3 mg vitamin K (Synkavite), 20 meq K^+^ (as KCl); and (b) an intra‐venous solution (0.6 mL/h) containing 26 mL H_2_O, 16 mL 0.5 N lactated Ringer, 20% glucose (Baxter), 0.32 g oxacillin Na for the initial 24 hours; then 8.5% Travasol amino acids (Baxter) and 20% Intralipid (Kabi) were added subsequently to provide adequate nutrients,^48^, ^49^ and 40 mg/kg/day BGP‐15 (NGene Research Laboratories, Inc). Body temperature, peripheral perfusion and oxygen saturation (measured continuously with an infrared probe in a hindlimb paw, MouseSTAT, Kent Scientific corp.) were monitored and maintained in the physiological range. The sham‐operated controls were anaesthetized with isoflurane, maintained in spontaneous breathing, received intra‐venous and intra‐arterial solutions, and killed within 2 hours of the initial isoflurane anaesthesia and surgery.

During surgery or any possible irritating manipulation, the anaesthetic isoflurane level, that is, the minimum alveolar concentration (MAC) was >1.5%, which maintains the following states: (a) the electroencephalogram (EEG) was synchronized and dominated by high‐voltage slow‐wave activity; (b) mean arterial pressure, 90‐100 mm Hg, heart rate maintained below 420 beats/min and (c) no evident EEG, blood pressure or heart rate responses to surgical manipulation. Isoflurane was delivered into the inspiratory gas stream by a precision mass‐flow controller. After the initial surgery, isoflurane was gradually lowered (over 1‐2 days) and maintained at MAC <0.5% during the remaining experimental period. Rats were ventilated through a coaxial tracheal cannula at 72 breaths/min with an inspiratory and expiratory ratio of 1:2 and a minute volume of 180‐200 mL and gas concentrations of 40% O_2_, 56,5% N_2_ and 3% CO_2_, delivered by a precision (volume drift <1%/wk) volumetric respirator. Airways pressure was monitored continuously as well as end‐tidal CO_2_ (EtCO_2_) and normocapnic condition maintained (EtCO_2_ = 37‐45 mm Hg) as well as normoxia (SpO_2_ >90%). Intermittent hyperinflations (six per hour at 19‐20 cm H_2_O) over a constant positive end‐expiratory pressure (PEEP = 1.5 cm H_2_O) were set to prevent atelectases. Post‐synaptic NMB was induced on the first day (150 µg α‐cobrotoxin) and maintained by continuous infusion (187 µg/day). Mechanical ventilation started after the NMB induction avoiding hypercapnia and hypoxaemia. Experiments were terminated after 5, 8 and 10 days. Female rats were preferred because of easier urine bladder catheterization for diuresis monitoring. The diuresis was maintained above 1 mL/h. In no case did animals show any signs of infections or septicaemia. The ethical committees at Uppsala University and Karolinska Institutet approved all aspects of this study. The material submitted conform with Good Publishing Practice in Physiology (Good publishing practice in physiology: Acta Physiol (Oxf). 2017 Dec;221(4):283‐284).

### 4.2 Muscle tissue, membrane permeabilization and single muscle fibre contractile measurements

At the end of the required duration of exposure to the ICU condition, the rats were killed, and the weights of the rats were recorded to measure the change in body weight for each group. In addition, upon dissection the soleus weight was recorded to check for comparison between different groups, and to calculate the ratio of soleus muscle to total body weight for each rat. The soleus muscle was then divided into three parts. One part of the muscle was quickly frozen in liquid propane cooled by liquid nitrogen and stored at −160°C for further analyses, and the same method was used for freezing the diaphragm for qPCR and protein analysis. Another part of the soleus and the diaphragm was placed in relaxing solution at 4°C and bundles of ~50 fibres were dissected free and tied with surgical silk to glass capillary tubes at slightly stretched lengths. The bundles were then treated with skinning solution (relaxing solution containing glycerol; 50:50 v/v) for 24 hours at 4°C, after which they were transferred to −20°C. Within 1‐2 weeks, the muscle bundles were treated with a cryoprotectant and snap frozen in liquid nitrogen‐chilled propane and stored at −180°C for long‐term storage. The final part of the soleus was fixed in 2.5% glutaraldehyde in 0.1 M cacodylic acid buffer at pH 7.4 and incubated at 4°C for at least 48 hours for subsequent transmission electron microscopy (TEM) analysis.

On the day of contractile measurements, bundles were transferred to a 2.0 M sucrose solution and subsequently incubated in solutions of decreasing sucrose concentrations (1.5‐0.5 M) for 30 minutes each and finally kept in a skinning solution at −20°C. A single fibre was removed from the muscle bundle and was placed between two connectors. One connector leads to a force transducer (model 400A, Aurora Scientific), and the other to a lever arm system (model 308B, Aurora Scientific). The two extremities of the fibre were attached to the connectors as previously described.^50^ The apparatus was mounted on the stage of an inverted microscope (model IX70; Olympus). While the fibre segments were in relaxing solution, the sarcomere length was set to 2.65‐2.75 µm by adjusting the overall segment length and controlled during the experiment using a high‐speed video analysis system (model 901A HVSL, Aurora Scientific). The diameter of the fibre segment between the connectors was measured through the microscope at a magnification of ×320 with an image analysis system prior to the mechanical experiments. Fibre depth was measured by recording the vertical displacement of the microscope nosepiece while focusing on the top and bottom surfaces of the fibre. The focusing control of the microscope was used as a micrometre. Fibre CSA was calculated from the diameter and depth, assuming an elliptical circumference, and was corrected for the 20% swelling that is known to occur during skinning.^50^ Diameter and depth were measured at three different locations along the length of each fibre and the mean was considered representative of cell dimensions.

For the mechanical recording, relaxing and activating solutions contained (in mM) 4 Mg‐ATP, 1 free Mg^2+^, 20 imidazole, 7 EGTA, 14.5 creatine phosphate and KCl to adjust the ionic strength to 180 mM and pH 7.0. The concentrations of free Ca^2+^ were 10^−9^ M (relaxing solution) and 10^−4.5^ M (activating solution), expressed as pCa^2+^ (ie −log [Ca^2+^]). Apparent stability constants for Ca^2+^‐EGTA were corrected for temperature (15°C) and ionic strength (180 mM). The computer program from Fabiato^51^ was used to calculate the concentration of each metal, ligand and metal‐ligand complex. Immediately preceding each activation, the fibre was immersed for 10‐20 seconds in a solution with a reduced Ca^2+^‐EGTA buffering capacity. This solution is identical to the relaxing solution except that the EGTA concentration is reduced to 0.5 mM, which results in a more rapid attainment of steady force during subsequent activation.

Force was measured by the slack‐test procedure.^52^ This was calculated as the difference between the maximal steady‐state isometric force in activating solution and the resting force measured in the same segment while in the relaxing solution. Maximal force production was normalized to CSA (specific force, absolute force (P_0_)/CSA). For contractile measurements, strict acceptance criteria were applied. First, the sarcomere length was checked during the experiments, using a high‐speed video analysis system (model 901A HVSL, Aurora Scientific). A muscle fibre was accepted and included in the analyses: (a) if the sarcomere length of a single muscle fibre changed <0.10 µm between relaxation and maximum activation and (b) if maximal force changed <10% from first to seventh activation.^50^

### 4.3 Modified single fibre myosin in vitro motility assay for speed and force measurement

Unregulated actin was purified from rabbit skeletal muscle^53^ and fluorescently labelled with rhodamine‐phalloidin (Invitrogen). The modified single fibre myosin in vitro motility system for speed and force measurement has been described in detail elsewhere.^26^, ^27^, ^54^ In brief, a short muscle fibre segment (1‐2 mm) was placed on a glass slide between two strips of grease, and a cover slip placed on top, creating a flow cell of ~2 µL. Myosin was extracted from the fibre segment through addition of a high‐salt buffer (0.5 M KCI, 25 mM HEPES, 4 mM MgCl_2_ and 4 mM EGTA, and the pH value was adjusted to 7.6 before adding 2 mM ATP and 1% β‐mercaptoethanol). After 30 minutes incubation on ice, a low‐salt buffer (25 mM KCl, 25 mM HEPES, 4 mM MgCl_2_ and 1 mM EGTA, with the pH value adjusted to 7.6 before adding 1% β‐mercaptoethanol) was applied, followed by BSA (1 mg/mL). Non‐functional myosin molecules were blocked with fragmented F‐actin, and rhodamine‐phalloidin–labelled actin filaments were subsequently infused into the flow cell, followed by motility buffer to initiate the movement (2 mM ATP, 0.1 mg/mL glucose oxidase, 23 µg/mL catalase, 2.5 mg/mL glucose, and 0.4% methyl cellulose in low‐salt buffer). The pH of the buffers was adjusted with KOH, and the final ionic strength of the motility buffer was 71 mM. The flow cell was placed on the stage of an inverted epifluorescence microscope (model IX 70; Olympus) and thermostatically controlled at 25°C. Actin movements were filmed with an image‐intensified SIT camera (SIT 66; DAGE‐MIT) and recorded on videotape.

The unit concentration (0.1 μg/mL in this study) of α‐actinin (A9776, Sigma) dissolved in the low‐salt buffer was chosen as the lowest concentration that would reduce the number of moving actin filaments. Multiples of this concentration were added to the flow cell over a fourfold range of α‐actinin concentration, from 0.1 to 0.4 μg/mL. A specific area on the glass slide was defined by a UV marking pen (Chr Winther‐Sörensen AB) before creating the flow cell. After identifying an organized moving filament, 3 μL α‐actinin was applied to the flow cell, then incubated for 1 minute and added motility buffer (2 mM ATP, 0.1 mg/mL glucose oxidase, 23 µg/mL catalase, 2.5 mg/mL glucose and 0.4% methyl cellulose in low‐salt buffer). Moving filaments within the defined area, with constant myosin concentration, were recorded for 12 seconds to allow measurement of the number and speed of actin filaments at the different α‐actinin concentrations. The duration was long enough for reliable measurements, but short enough to minimize the risk of photo‐bleaching interference.

For speed analysis, we selected 20 actin filaments that were moving at constant speed in an oriented motion from each single fibre preparation. Recordings and analysis were only performed from preparations in which >90% of the filaments moved bi‐directionally. A filament was tracked from the centre of mass, and the speed was calculated from 10 frames at an acquisition rate of five or one frame(s) per second, depending on the fibre type, using an image analysis package (Image‐pro Plus Version 6.0, Media Cybernetics).^55^ The mean speed of the 20 filaments was calculated. For force analysis, the negative regression line was plotted between the relative fraction of moving actin filaments propelled by myosin and α‐actinin concentration, such that the number of moving actin filaments in a specific volume in the flow cell relative to the number of moving filaments without the addition of α‐actinin are plotted against alpha‐actinin concentrations, and the *x*‐axis intercept was used as an index to evaluate the force‐generating capacity of myosin.^26^

### 4.4 The affinity of myosin ATPase from the single fibre preparation

The Michaelis‐Menten constant (Km), reflecting the affinity of myosin ATPase to ATP equilibrium,^56^ was evaluated using quantum dot (QD)‐mediated nanothermometry.^57^ Myosin was extracted from 5 to 6 mm long fibre segments in 10 µL high‐salt buffer for 30 minutes and subsequently added to 6 wells (1 µL per each) containing 30 µL KS buffer on the black 384‐well microtitre plate, which was followed by the addition of 1 µL diluted Cadmium telluride core‐type QDs (1 mg/mL) (Sigma‐Aldrich) and motility buffer (30 µL). Motility buffers were prepared with six different ATP concentrations and concentrations were optimized according to reaction performance. QD fluorescence signal was recorded (fluorescence spectrophotometer, TECAN, Infinite M200). The excitation wavelength was fixed at 350 nm, and emission collected at 540 nm. The fluorescence was recorded every 15 seconds during 300 seconds and the negative hyperbolic curves of the fluorescent signal were created. The linear slope during the initial period of 90 seconds was calculated, that is, the initial velocity of the reaction. Measurements were repeated six times at different ATP concentrations, and the Km (indicating the affinity of myosin ATPase to ATP) was calculated from Lineweaver‐Burk equation converted from Michaelis‐Menten equation.

### 4.5 Myosin: actin ratio

Actin and myosin contents were quantified on a 12% SDS‐PAGE gel. The acrylamide concentration was 4% (W/V) in the stacking gel and 12% in the running gel, and the gel matrix included 10% glycerol. Electrophoresis was performed at 32.0 mA for 5 hours with a Tris‐glycine electrode buffer (pH 8.3) at 15°C (SE 600 vertical slab gel unit; Hoefer Scientific Instruments). The gels were stained using SimplyBlue SafeStain (Invitrogen) and subsequently scanned in a soft laser densitometer (Molecular Dynamics) with a high spatial resolution (50 µm pixel spacing) and 4096 optical density levels. The volume integration function was used to quantify the amount of protein and the results were presented as myosin: actin ratio.

### 4.6 Mass spectrometry driven proteomics

#### 4.6.1 In‐gel digestion

The type I, IIa, IIx and, in some instances, IIb, MyHC isoforms were separated on a 6% SDS‐PAGE gel. The bands were cut out and stored at −80°C before the sample preparation took place. A thawed gel piece was first washed with 100 mm NH_4_HCO_3_ and then reduced with 10 mm dithiothreitol at 56°C for 30 minutes. Alkylation was initiated by adding iodoacetamide in 2.2‐fold excess and the sample was then incubated in darkness at room temperature for 40 minutes. The solution was removed, and the gel piece was rinsed with 50 mm NH_4_HCO_3_ in 50% acetonitrile (ACN). The solution was removed, and the gel piece was rinsed with ACN. The gel piece was dried at 50°C before the addition of 0.025 μg mL^−1^ trypsin. The gel piece was allowed to soak at 8°C for 1 hour before the addition of 100 mm NH_4_HCO_3_. The sample was then kept at 37°C overnight. On the following morning, the sample was sonicated for 5 minutes and the liquid collected and dried under vacuum. The peptides were stored at −20°C until further analysis, when they were dissolved in 0.1% trifluoroacetic acid.

#### 4.6.2 Liquid chromatography (LC)/mass spectrometry (MS) analysis

The peptides were analysed using an LTQ‐Orbitrap Velos Pro (Thermo Scientific). Separation of the peptides was performed using an EASY‐nLC II system (Thermo Scientific). An EASY column, 2 cm, i.d. 100, 5 μm, C18‐A1 (Thermo Scientific) was used as a pre‐column and an EASY column, 10 cm, i.d. 75, 3 μm C18‐A2 (Thermo Scientific) was used as an analytical column. The mass analyser and the separation system were controlled using Tune 2.6.0 and Xcalibur 2.1. The sample was loaded on the pre‐column using Solvent A (0.1% formic acid) with 2% Solvent B (0.1% formic acid in acetonitrile). After the loading, 2 minutes of 2% B using a flow rate of 200 nL min^−1^ followed. The first step of the gradient was an increase from 2% Solvent B to 35% Solvent B during 15 minutes. The second step was an increase from 35% Solvent B to 50% Solvent B during 5 minutes. The third step was an increase from 50% Solvent B to 80% Solvent B during 5 minutes. The flow rate was then increased from 200 nL min^−1^ to 400 nL min^−1^ during 3 minutes, and 80% Solvent B with a flow rate of 400 nL min^−1^ was then kept for 8 minutes.

For the MS analysis, a survey scan was performed for m/z from 400 to 2000 at a resolution of 60 000 using the Orbitrap. Following this event, 10 data‐dependent MS/MS spectra were recorded using the 10 most intense ions from the survey scan. This MS/MS analysis was performed using the linear ion trap. The precursor ions were isolated within a 1 Da window and fragmented by collision‐induced dissociation with 35% normalized collision energy. The activation time was set to 10 ms and *q* = 0.25. Dynamic exclusion was set to 30 seconds and the exclusion mass width was set to 5 p.p.m. relative to the reference mass.

#### 4.6.3 Data analysis

The acquired data (.RAW‐files) were processed using Proteome Discoverer (Thermo Scientific, version 1.4.0.288), where SEQUEST was used to search the results against the Uniprot‐Swissprot database for Rattus norvegicus taxonomy. For all searches, the enzyme specificity was trypsin; two missed cleavages were allowed; error tolerances of 0.02 and 0.7 were set for the survey scan and the MS/MS analysis, respectively; carbamidomethylation (C) was set as a static modification; oxidation (M) and deamidation (N, Q) were set as variable modifications. In order to search for many post‐translational modifications (PTMs), the processing was performed in four blocks with following variable modifications: (a) methylation (C‐terminal, D, E), carbamylation (K); (b) oxidation (F, H, K, P), phosphorylation (S, T, Y); (c) oxidation (D, N, R, W, Y) and (d) methylation (C‐terminal), acetylation (K, S), nitration (W, Y). The search results from these blocks were merged and validated using Percolator (embedded in Proteome Discoverer), in which the target false discovery rate was set to 0.01 (strict) and 0.05 (relaxed). Out of the resulting peptides, only those with medium or high confidence were considered for further processing. All peptides with found PTMs were further processed as follows. First, all peptides containing only carbamidomethylations and/or methionine oxidations and/or carbamylations, as well as all peptides belonging to proteins other than myosin, were excluded. Second, only PTM sites reported in at least three CMV samples and absent in controls were taken into account. Third, all remaining peptides were verified, that is, their mass spectra were manually checked for PTM confirmation. The latter was done based on knowledge of basic theory of peptide fragmentation, as well as considering possible misinterpretations (description of the details can be found in^58^). Only fully confirmed (with unambiguous MS/MS spectra) PTMs were reported in the results.

### 4.7 Transmission electron microscopy (TEM)

Eight soleus muscle samples from rats were prepared for TEM analysis: two control, two in 5d group, two in 5d + BGP, one in 10d group and one in 10d + BGP group. After fixation with 2.5% glutaraldehyde and incubated overnight at +4°C, the soleus samples were post‐fixed with 1% osmium tetraoxide for 3 hours at room temperature. Then, the samples were dehydrated using increasing concentration of ethanol (70%, 95% and 99% three times each). After dehydration, the samples were infiltrated with propylene oxide for 10 minutes, and then infiltrated with increasing concentration (50% and 100%) of resin (agar 100 resin, agar scientific) in propylene oxide over 24 hours. Later, the samples were placed in the desired moulds with 100% resin and incubated at 60°C for 3 days. To insure the orientation and quality of the sample sections, 1‐µm sections were stained with toluidine blue and viewed using normal light microscope. Ultrathin sections of 60‐80 nm were cut using diamond knife of ultramicrotome. Sections were transferred into copper grids, and stained with uranyl acetate and lead citrate. Finally, the ultrathin sections were examined on a transmission electron microscope (JEOL 1230; JEOL Ltd.) operated at 100 kV, and digital micrographs were taken using Gatan multiscan (model 791, Gatan) and Gatan digital software version 3.6.4 (Gatan).

To analyse both IMF and SS mitochondria, 10 electron micrographs for 5 random fibres in the longitudinal orientation were obtained at 10.000× magnification. From each fibre, two random images for IMF and SS mitochondria were captured, allowing the analysis of ≈40 IMF and 25 SS mitochondria. For each rat, a minimum of 200 IMF and 125 SS mitochondria were analysed. Each electron micrograph was then analysed using Image J (1.48 version, national institute of health, Bethesda, MD) by manually tracing the outlines of IMF and SS mitochondria,^59^ and then taking the ratio between the normal and abnormal mitochondrial morphology for each micrograph.

### 4.8 Total RNA isolation and quantification

Total RNA was extracted from frozen soleus and diaphragm muscle tissue (10‐30 mg) using QiagenRNeasy® Mini Kit (Qiagen, Inc). Muscle tissue was homogenized using a motor homogenizer (Eurostar Digital, IKAWerke). QIAshredder™ columns (Qiagen Inc) were used to disrupt DNA. Total RNA was eluted from RNeasy® Mini columns with 30 µL of RNase‐free water. RNA concentrations were then quantified using nanodrop 1000 spectrophotometer (Thermofisher Scientific).

### 4.9 Quantitative real‐time PCR

qPCR was used to quantify the mRNA levels for rat PGC‐1α, MFN1, MFN2, OPA1, DRP1, MyHCI, MyHCIIa, MyHCIIx and actin. One hundred nanograms of total RNA from soleus samples was reverse transcribed to cDNA using Qscript cDNA supermix (Quanta Biosciences). cDNA was amplified in duplicate using MyiQ™ single colour real‐time PCR detection system (Bio‐Rad Laboratories, Inc). The thermal cycling conditions include 95°C for 10 minutes, followed by 50 cycles of a two‐step PCR with denaturation at 95°C for 15 seconds and a combined annealing and extension step at 60°C for 1 minute. Each reaction was performed in a 20 µL volume with 0.4 µM of each primer and 0.2 µM of SYBR Green. TaqMan primers were designed using the software Primer Express® (Applied Biosystems). Primer sequences were provided on the supplement Table S1.

### 4.10 Statistical analyses

Statistical analysis was performed using the Sigma Plot 13 software. One‐way analysis of variance (ANOVA) and the Tukey post hoc analysis were used when comparing multiple groups. If the analysed data measures failed the normality testing, ANOVA on ranks was performed. Unpaired student *t* test was used when comparing the effect of BGP‐15 after 5, 8 and 10 days of CMV *P* < .05 was considered statistically significant. Unless stated, data are presented as means ± standard error of the means.

## CONFLICT OF INTEREST

We declare no conflict of interest.

## Data Availability

The data that support the findings of this study are available from the corresponding author upon reasonable request.
