# Supplementary material for: Chaperone co‐inducer BGP‐15 mitigates early contractile dysfunction of the soleus muscle in a rat ICU model
Source: Acta Physiol (Oxf). 2019 Dec 18;229(1):e13425. doi: 10.1111/apha.13425 (PMC7187345; doi:10.1111/apha.13425)
Supplement: Supplementary file 1 [file APHA-229-e13425-s001.pdf]

Supplementary material

S1. Real-Time PCR Primer List

Transcripts	Primer FWD 5´- 3	Primer REV 5´- 3
**GAPDH**	AACCCATCACCATCTTCCAG	GTGGTTCACACCCATCACAA
**PGC 1α**	TGATGTGAATGACCTGGACACAGACA	GCTCATTGTTGTACTGGTTGGATATG
**MFN1**	CTGCGATCTTCGGCCAGTTA	AGCCCTGTATCTCCACCAGAT
**MFN2**	GACTCCAGCCATGTCCATGAT	GGCAGTGACAAAGTGCTTGAG
**OPA1**	TCTTCACTGCGGGTACACCT	TTCTCCTTCTCCAAACGCTCC
**DRP1**	ATGGTGGTCAGGAACCGACA	GCAAATTGACAGCATGGCCT
**MyHCI**	CTCGCTCCCTCAGCACAGA	CTGCTCAGTCAAGTCGGAGATCT
**MyHCIIa**	AAACCCTGAAGCGAGAGAACAA	TCATGGATACGTTTCCCTCCTT
**MyHCIIx**	CAAAGGTGAAATCCTACAAGAGACAA	TGGATCCTCCGGAATTTGG
Atrogin-1	GTCCAGAGAGTCGGCAAGTC	GTAGCCGGTCTTCACTGAGC
**MuRF1**	GAGAACCTGGAGAAGCAGCT	CCGCGGTTGGTCCAGTAG
**SMART**	ACCATGGCGTCGGTAGCGGGGACA	CTCGGCTGTGTCCTCCTTTGCACTG
**Fbxo31**	CCATACGGAGGACTGCTGA	GTACATCCACCCGATGATGA
**Actin**	AGGTCATCACCATCGGCAAT	AAGGAAGGCTGGAAGAGCGT
